# High performance silicon electrode enabled by titanicone coating

**DOI:** 10.1038/s41598-021-04105-x

**Published:** 2022-01-07

**Authors:** Zahilia Cabán Huertas, Daniel Settipani, Cristina Flox, Joan Ramon Morante, Tanja Kallio, Jordi Jacas Biendicho

**Affiliations:** 1 Aalto University, Kemistintie 1, 02150 Espoo, Finland; 2 Catalonia Institute for Energy Research, Jardins de les Dones de Negre 1, 2ª p., 08930 Barcelona, Spain; 3 Faculty of Physics, University of Barcelona, Marti I Franques, 1, 08028 Barcelona, Spain

**Keywords:** Chemistry, Engineering, Materials science

## Abstract

This paper presents the electrochemical performance and characterization of nano Si electrodes coated with titanicone (TiGL) as an anode for Li ion batteries (LIBs). Atomic layer deposition (ALD) of the metal combined with the molecular layer deposition (MLD) of the organic precursor is used to prepare coated electrodes at different temperatures with improved performance compared to the uncoated Si electrode. Coated electrodes prepared at 150 °C deliver the highest capacity and best current response of 1800 mAh g^−1^ at 0.1 C and 150 mAh g^−1^ at 20 C. This represented a substantial improvement compared to the Si baseline which delivers a capacity of 1100 mAh g^−1^ at 0.1 C but fails to deliver capacity at 20 C. Moreover, the optimized coated electrode shows an outstanding capacity of 1200 mAh g^−1^ at 1 C for 350 cycles with a capacity retention of 93%. The improved discharge capacity, electrode efficiencies, rate capability and electrochemical stability for the Si-based electrode presented in this manuscript are directly correlated to the optimized TiGL coating layer deposited by the ALD/MLD processes, which enhances lithium kinetics and electronic conductivity as demonstrated by equivalent circuit analysis of low frequency impedance data and conductivity measurements. The coating strategy also stabilizes SEI film formation with better Coulombic efficiencies (CE) and improves long cycling stability by reducing capacity lost.

## Introduction

The demands for developing advanced energy storage devices have dramatically increased during the past decade. One reason for this is that our society is living a communication revolution; another important motive is the implementation of the electric vehicle in a decarbonized society. Due to the environmental issues affecting our planet, more efficient energy storage technologies are needed. Therefore, batteries will play an important role in the new renewable energy based smart grid development and off the grid applications. The development of high energy density and fast charge/discharge lithium batteries requires new materials. Commercial batteries use graphite and layered oxides with cell energy densities up to 300 Wh kg^−1^^1^ which are lower than the requirements claimed for having a competitive autonomy in an electric battery based mobility^2^.

Silicon is a promising material as a negative electrode for LIBs. It can store almost 4 mol of Li per mol of Si (Li_15_Si_4_) leading to a theoretical volumetric capacity of 2190 mAh L^−1^^3^ which is higher than the graphite one i.e. 837 mAh L^−1^^4^. Additionally, Si has a low discharge potential of 0.05 V vs. Li/Li^+^^5^ and it is an abundant element on the Earth’s crust; its low cost and the well-developed manufacturing infrastructure make this material a promising candidate for high energy (next generation) LIBs. However, there are some complications during silicon performance. It exhibits a large volume expansion upon Li insertion and removal of approximately 300% of its original size^6^ which leads to severe electrode degradation, loss of electric contact and continuous side-effect reactions and, eventually, to an increased cell resistance, low rate-capability and a low Coulombic efficiency (CE)^7^. Different approaches have been proposed to overcome silicon limitations^8^. For instance, the use of nanoparticles with a critical particle diameter below 150 nm to reduce particle cracking upon first lithiation^9^, formulation of new and effective binder compositions to better compensate for the volume expansion of the silicon^10^, synthesis of silicon nanostructures e.g. core–shell^11^, nanowires^12^ or nanotubes^13^, with improved performance compared to micro-scale powders, and fabrication of composite materials with carbon to account for the volumetric changes while preserving electrical contact^14^. Alternatively, silicon can also be prelitiated (Li_x_Si_y_) to avoid its large volume expansion on initial cycles but these phases appear to be highly unstable at room temperature^15^.

A more practical and rational way to mitigate the aforementioned issues that have impeded a performant Si electrode is based on surface modifications by using ALD combined with the more powerful MLD. By combining these two techniques, the substrate is exposed to metal and organic precursors that go through a self-limiting reaction resulting in the deposition of a hybrid inorganic–organic film known as metalocone. MLD yield a coating with a lot of attractive properties such as precise structure and thickness control and conformal high aspect ratio structures. Recent studies conducted by Abdulagatov et al. demonstrate that combining TiCl_4_-glycerol (GL) results in a film with improved mechanical properties compared to TiCl_4_-ethylene glycol (EG)^16,17^. This approach can be used to produce flexible coatings to control the chemical reactivity and volume expansion of silicon; an example is alucone coating which provides significant improvements in cycling stability, rate capability, and CE for nano-Si composite electrodes^18^ up to 2000 mAh g^−1^ at 0.05 C. Other interesting example is the use of zincone which improves discharge capacity to 1741 mAh g^−1^ at 2000 mAg^−1^ and that is related to the formation of a stable LiF-rich SEI layer for the coated sample^19^.

However, MLD has one main disadvantage associated with the homo-bifunctional reaction sequence between precursors and a surface which limits the number of active surface sites. Thus, the double reactions hinder the growth of the thin layers. The use of glycerol eliminates this problem since it is a homo-trifunctional precursor^20^. Glycerol characteristics will lead to more bridging between the polymer chains. MLD requirements for the precursors are sufficient vapor pressure, reactivity, and stability at the reaction temperature to ensure feasible film growth^21^. Many organic precursors exhibit low vapor pressures at room temperature, and it is thus mandatory to heat them to achieve a sufficient precursor supply. Therefore, finding organic compounds which would fulfill the requirements for MLD reaction is not simple^22^.

The use of Ti-based coatings to enhance the performance of Si electrodes has already been discussed by others^23–25^. A recent example is the use of TiO_2_ as amorphous layer to encapsulate Si particles delivering high capacity of 2804 mAh g^−1^ at 1 Ag^−1^ for the first cycle. This improvement was attributed to the hollow core–shell nanostructure of the material^23^. Additionally, Ti coating enhances the electrical conductivity of Si electrodes for samples prepared by electron beam physical vapor technique^24^. Also titanicone has been used to enhance the electrochemical performance of N-CNTs towards Li storage^25^.

Consequently, our purpose is to develop a titanicone coating to modify the surface of Si and improve, in turn, the electrochemical performance of the anode for LIBs. The optimized deposition process of titanicone using ALD/MLD techniques described in this paper opens attractive options for more advantageous commercialization of Si-based electrodes.

## Results

**Figure 1:**
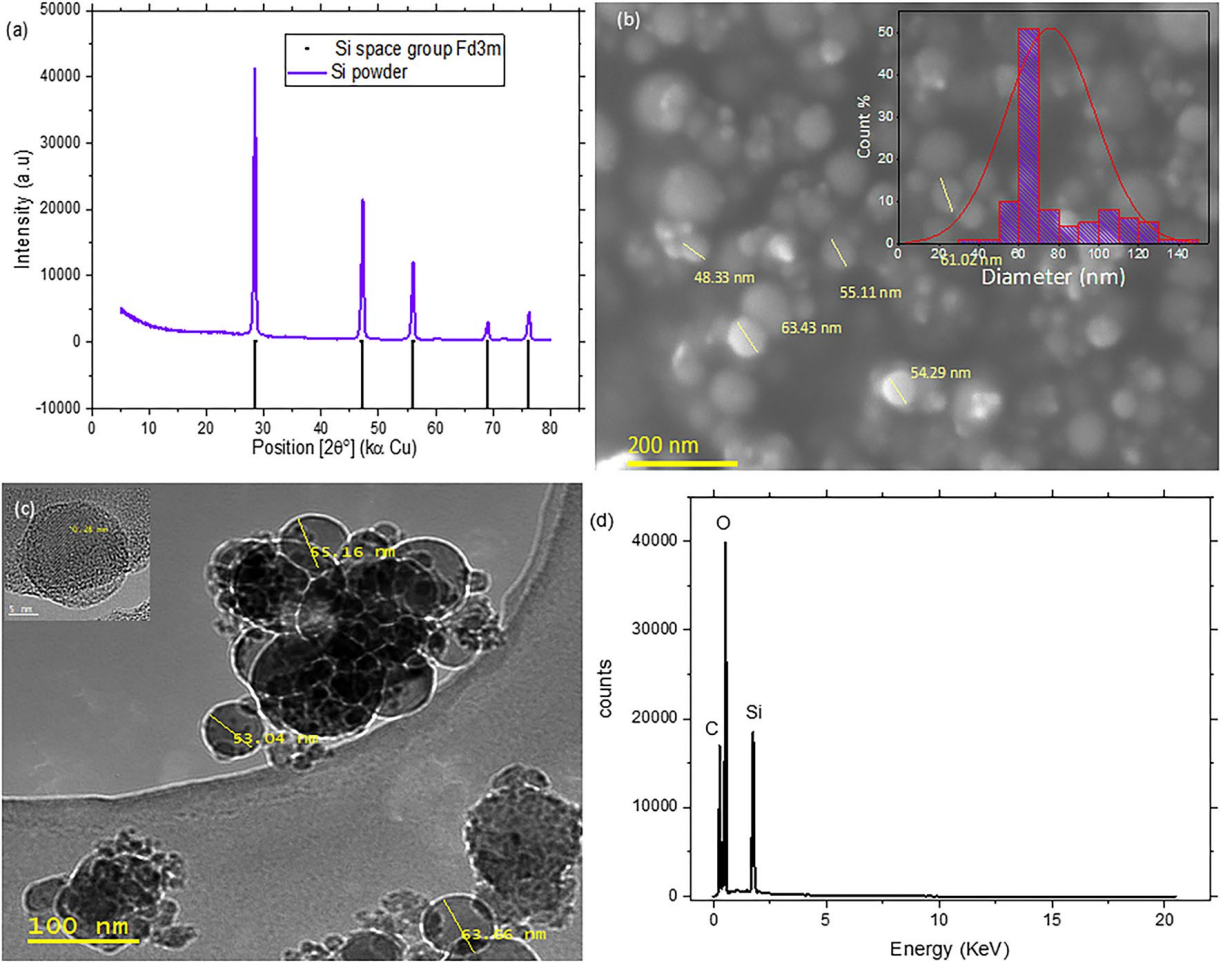
Structure, particle size/morphology and composition for the silicon (**a**) XRD pattern measured using Cu radiation, (**b**) a SEM image and histogram inset showing particle size distribution, (**c**) a TEM image and (**d**) EDX spectra.

Silicon powder from Alfa Aesar was first characterized to check its purity and particle size/morphology (Fig. 1). The XRD pattern shows a high-purity sample since all diffraction peaks correspond to silicon with a cubic crystal structure (S.G.: Fd-3 m) and *a* = 3.867(1) Å (Fig. 1a). A SEM image (Fig. 1b), shows particles of spherical shape with a diameter in the range of 40–145 nm, see histogram inset. The analysis shows that 50% of the particles have a diameter around 60 nm considering the diameter of 101 particles. The size and morphology of silicon particles was also investigated by TEM (Fig. 1c), which confirmed a homogenous distribution of nanoparticles. EDX spectrum (Fig. 1d), shows oxygen corresponding to the native oxide layer covering the nanoparticles while the C signal is related to substrate used for the SEM analysis.

**Figure 2:**
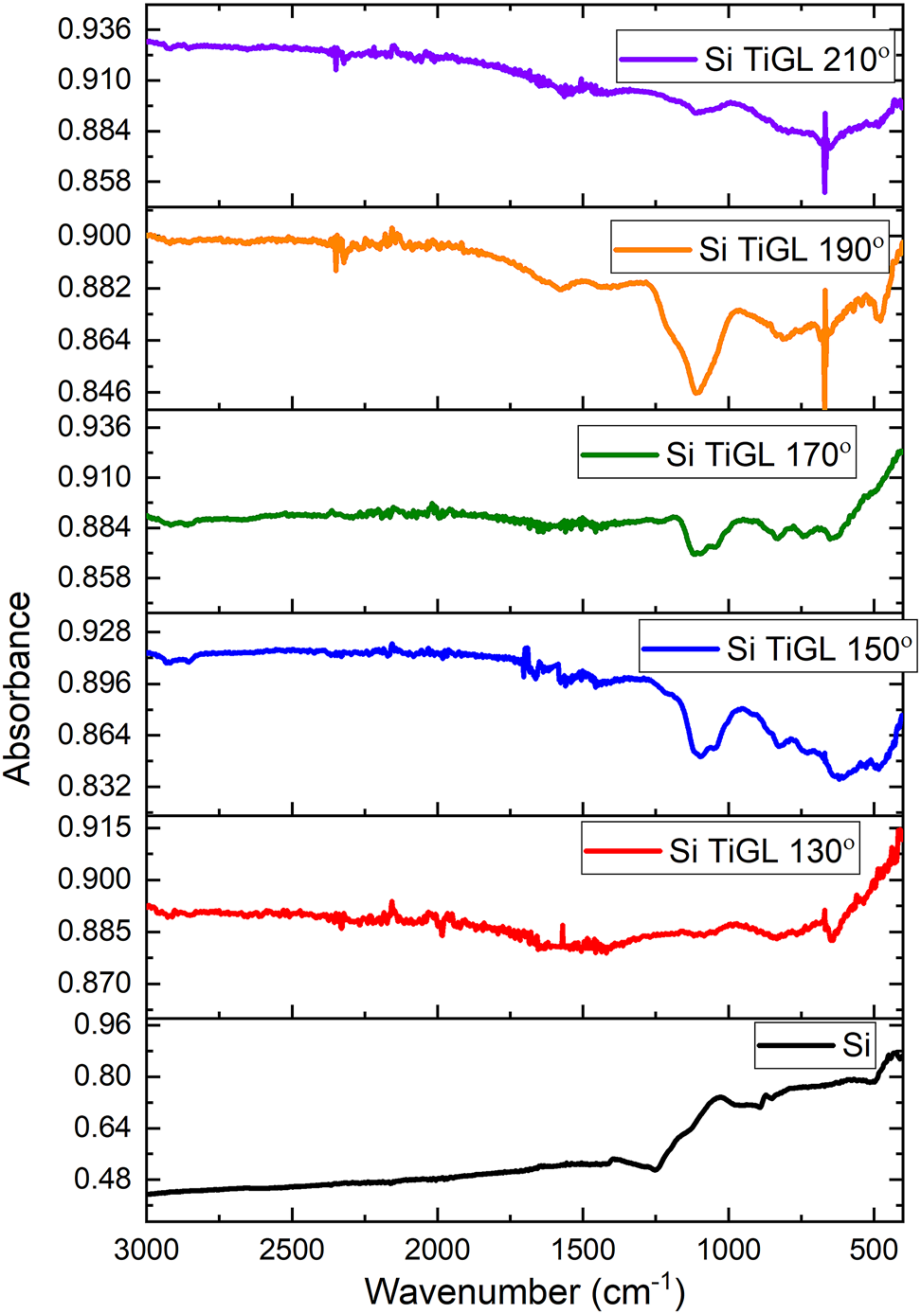
FTIR spectra of Si baseline and Si TiGL 130, 150, 170, 190 and 210 samples.

Si electrodes fabricated using a doctor blade were used as substrates for titanicone coating. ALD/MLD growth on a substrate typically depends on the deposition temperature, and hence it was optimized first. For this study, the number of cycles during titanicone deposition was fixed to 100 cycles, based on previous publications^26^. Temperatures 130 °C, 150 °C, 170 °C, 190 °C, and 210 °C were selected to carry out the deposition of TiGL on the Si electrode, table S1, and FTIR spectroscopy was first used to evaluate structure of the composite electrodes. Figure 2 shows the results obtained by FTIR and the corresponding analysis. The FTIR spectra recorded for the uncoated electrode (Si baseline) do not display any particular feature. However, coated electrodes (Si TiGL) show characteristic peaks of carbon and titanium vibrations. CH_2_ stretches originating from the glycerol molecule can be observed at wavenumbers 2928 cm^−1^ and 2870 cm^−1^, together with a more negligible absorption at 1248 cm^−1^. Other peaks associated with the GL molecules are C–C and C–O absorptions at 1135 cm^−1^ and 1086 cm^−1^. At low wavenumbers, vibration modes related to the Ti–O bonds are also present. More specifically, the 820 cm^−1^ sharp peaks are assigned to the Ti–O stretch mode. In the range 720–550 cm^−1^, absorptions bands are related to Ti–O–Ti bonds. The combination of both the carbon related groups and the Ti absorptions provides evidence of the successful deposition of a hybrid organic–inorganic film containing Ti and GL at temperatures 130 °C, 150 °C, 170 °C, and 190 °C. That is not the case for the sample prepared at 210 °C, which shows a decrease of the intensity for the associated FTIR peaks compared to the lower deposition temperatures used, especially those related to GL. According to previous reports^27^, high temperature applied to the MLD process does not yield high-quality thin films since the number of reactive sites and reaction mechanism is affected.

Coated electrodes were then characterized by SEM/EDX as presented in Figure S1 in Supporting Information for Si TiGL 150. Samples Si TiGL 130, 150, 170, and 190 showed a homogeneous distribution of titanium with no changes on the particle morphology with respect to the Si baseline. Also, there were no differences between diffraction patterns collected before and after coating (Fig. S2). Peaks were identified as Si and Cu, the last corresponding to the current collector. Cu peaks were index as F m-3 m (225).

**Figure 3:**
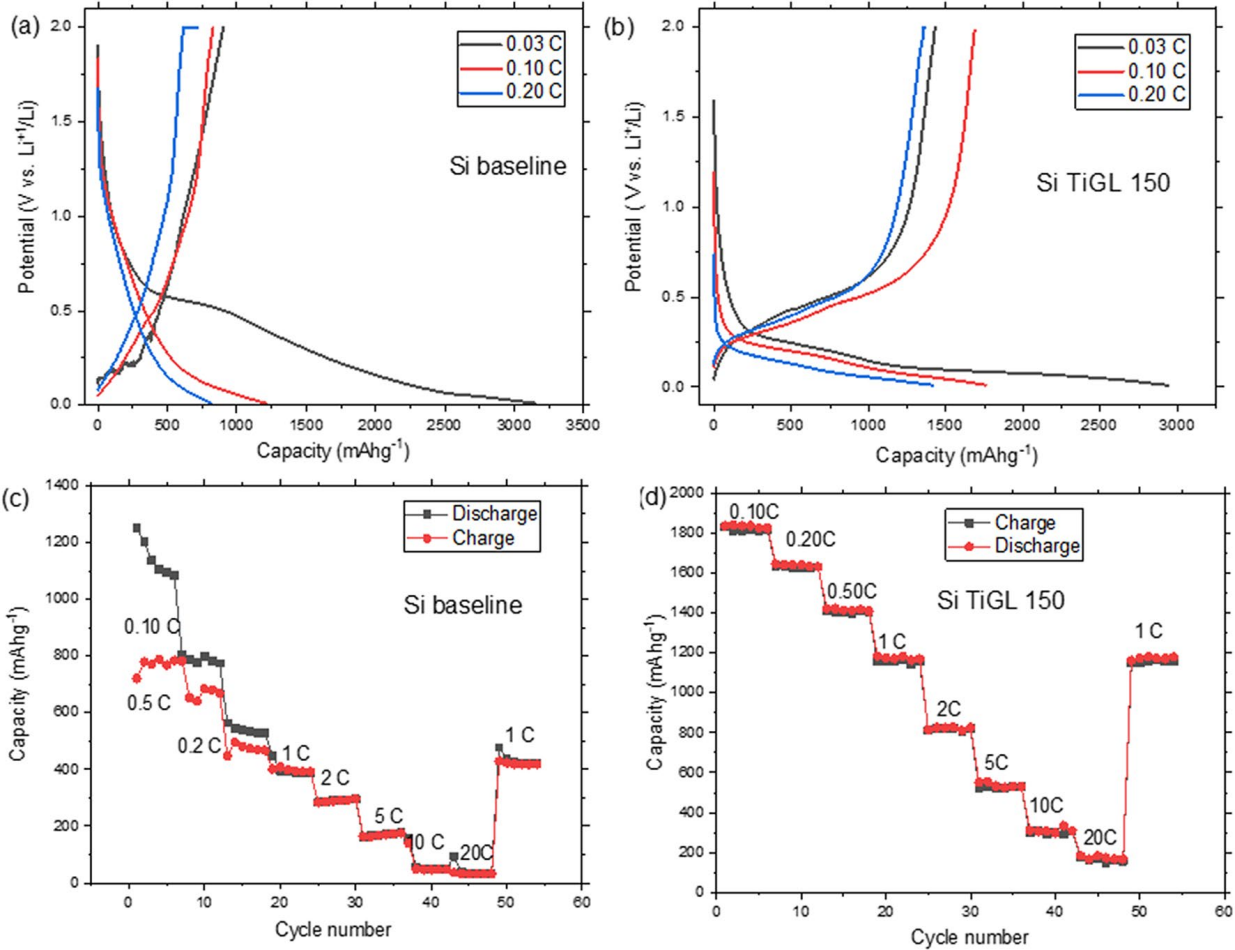
Electrochemical tests (**a**) and (**b**) charge/discharge profiles and (**c**) and (**d**) rate-capability tests for Si baseline and Si TiGL 150, respectively.

The electrochemical properties of the electrodes were evaluated in half-cells using different electrochemical tests. Electrodes were first activated at 0.03 C. Figure 3a,b show the activation profile (cycle 1) and characteristic charge/discharge curves for Si baseline and Si TiGL 150, respectively, as black 0.03 C, red 0.10 C and blue 0.20 C lines. Electrodes show typical charge/discharge profiles associated to Si reduction/oxidation with a low CE during the activation cycle (black lines) indicating that lithium is consumed from the electrolyte in irreversible reactions suggesting SEI formation. Si baseline shows the lowest CE in agreement with reported results for nanosized powders^28,29^ while for certain coated samples CE is significantly improved, for instance CE for Si TiGL 150 is 86% and 84% for Si TiGL 190 for cycle 2 activation, see Table S2 (Supporting Information) SI. This indicates sample coating reduces side reactions and promotes the formation of a more stable SEI film. After activation, the CE of the Si electrode at 0.1 C is 57% and Si TiGL 150, 170 and 190 show the highest efficiency among the samples in the range of 95%.

For rate capability tests, results are shown in Fig. 3c,d for Si baseline and Si TiGL 150. Si baseline shows discharge capacities of 1100, 800, 550, 400, 300 and 200 mAh g^−1^ at 0.1, 0.2, 0.5, 1, 2 and 5 C, respectively. At higher C-rates of 10 20 C, the electrode fails to deliver any significant capacity. On the other hand, Si TiGL 150 delivers high (and stable) discharge capacities: 1800, 1600, 1400, 1100, 800, 500 mAh g^−1^ at 0.1, 0.2, 0.5, 1, 2 and 5 C, respectively, which are in the range of best performing Si electrode materials reported up to date^30,31^. The performance at 10 C and 20 C rates is also significant delivering 300 and 150 mAh g^−1^, respectively. For these several C-rates tested, the CEs are always close to 100% indicating no side reactions at the electrodes. Results from Si TiGL 150 are best performing ones among all coated samples.

**Figure 4:**
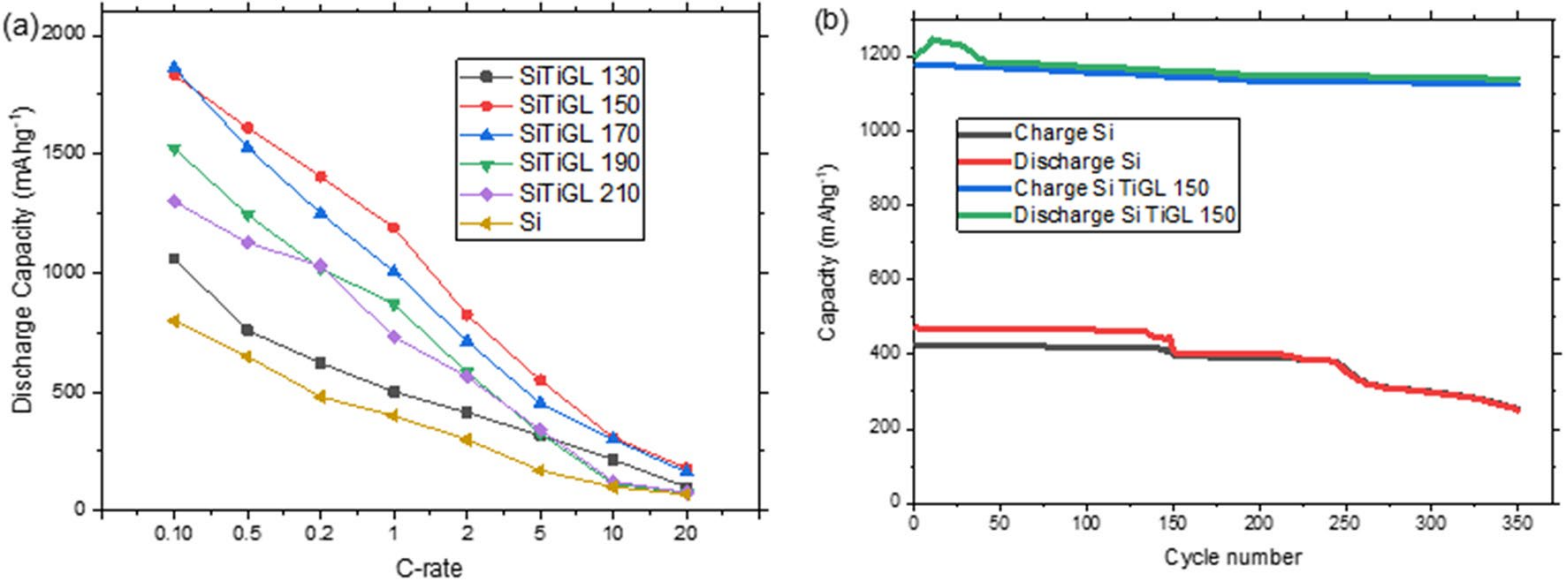
Electrochemical tests (**a**) C-rate test for all samples and (**b**) capacity retention measured at 1 C for Si baseline and Si TiGL 150.

Figure 4a shows a comparison between the Si TiGL samples as a function of C-rate. Si TiGL 130 shows similar performance to Si baseline and Si TiGL 170, 190 and 210 deliver lower capacities than Si TiGL 150 at all C-rates tested. From this, we can consider that synthesis conditions for titanicone coating on Si electrode should be adjusted to 150 °C using TiCl_4_ and glycerol as precursor and reactant, respectively, during the ALD/MLD deposition process. As for this deposition temperature, the electrode shows an enhanced kinetic performance which affords high capacity at C-rates up to 20 C. To further evaluate the performance of Si TiGL 150, a long-term cycling tests at 1 C up to 350 cycles was performed for this selected coated sample and Si baseline and results are presented in Fig. 4b. Discharge capacity for Si baseline is in the range of reported Si based electrodes using nano-scale powders^32^ delivering 400 mAh g^−1^ on initial cycling and then the capacity decreases in a step-wise manner down to 250 mAh g^−1^ with a capacity retention of 53%. Besides, Si TiGL 150 delivers a stable capacity of 1180 mAh g^−1^ with a 94% capacity retention up to cycle 350. Similar stability has been reported for alucone coating^18^. The enhanced performance was attributed to a better structural integrity of the coated electrode compared to bare silicon, preserving intimate contact between carbon and active particles upon lithiation, as evidenced by post-mortem studies^18^.

To get further insights into the kinetics of the Si TiGL electrodes, EIS and 4-point conductivity measurements were conducted on the selected samples. Figure S3 shows the current vs voltage plots for a Si wafer coated with TiGL (as reference), Si baseline and Si TiGL 150, respectively. The conductivity of the Si wafer coated with TiGL is 0.055 S cm^−1^, and conductivities of Si baseline and Si TiGL 150 are 0.00155 S cm^−1^ and 0.00799 S cm^−1^. So the electrical conductivity is increased for the Si TiGL 150 electrode.

**Figure 5:**
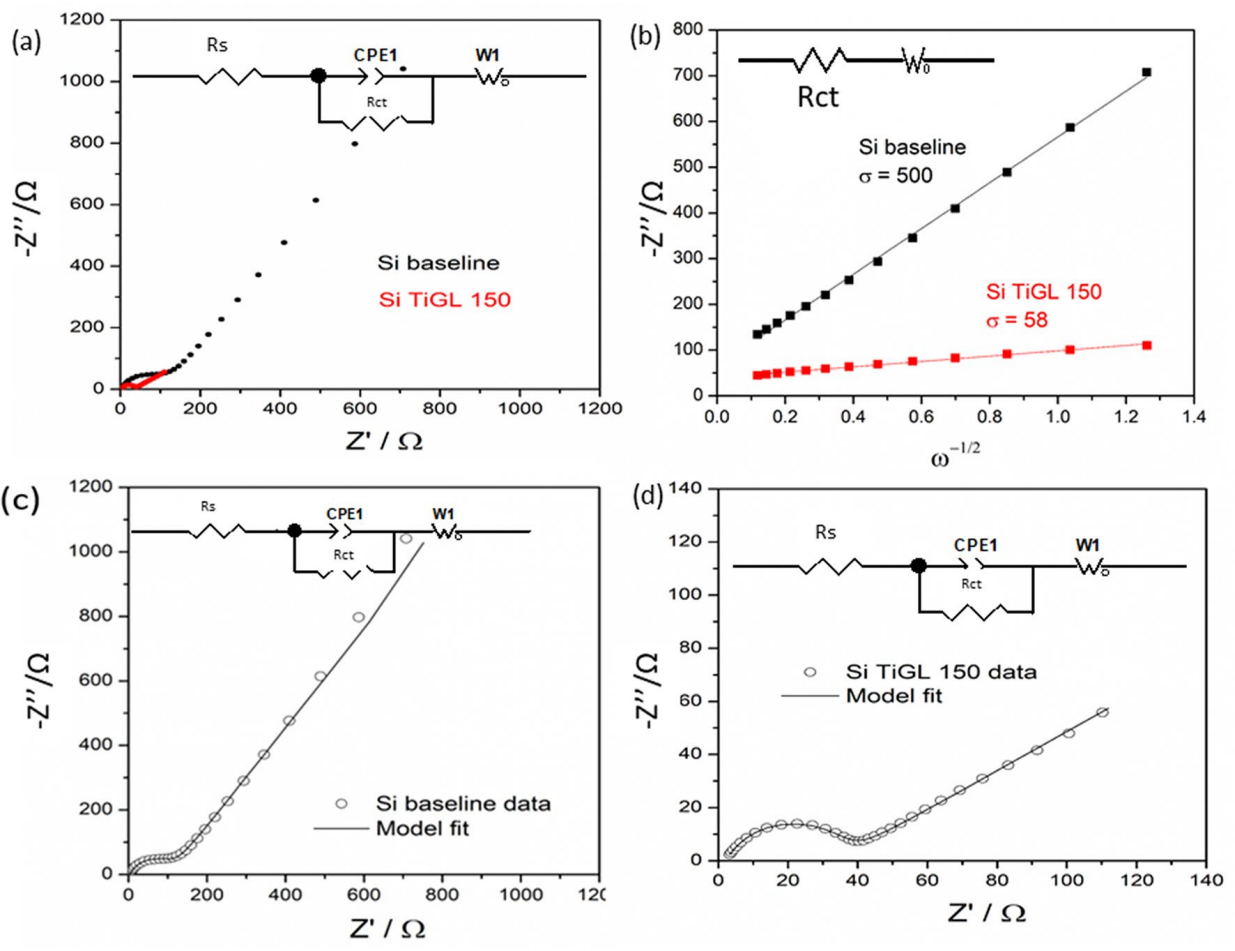
Impedance data presented in the form (**a**) Nyquist plots, (**b**) Z′ vs *ω*^−0.5^ plots and fitting results using Randles circuit inset, and Si baseline and Si TiGL 150 data fitting using the equivalent circuit inset in (**c**) and (**d**), after activation respectively.

Figure 5a shows Nyquist plots, b shows Z′ vs *ω*^−0.5^ plots and fitting results for c shows Si baseline and d Si TiGL 150 using the equivalent circuit inset. Impedance plots presented in the form of Z′ vs -Z″ (Fig. 5a), are characterized by a non-ideal semicircle at high frequency and a low frequency tail. The shape and slope of the tail is related to a frequency range where the kinetics of the system are almost entirely limited by the rate of the chemical diffusion process in the host material. The capacitance associated to each sample semicircle is in the range of 20 µF but their charge-transfer resistance (R_CT_) varies slightly being 100 Ω for Si baseline and 30 Ω for Si TiGL 150. Low frequency data presented in the form of Z′ vs *ω*^−0.5^ (Fig. 5b), show straight lines corresponding to R_CT_ and Warburg element in series, inset. The Z’ axis intercept is R_CT_ and the slope (or Warburg coefficient σ) is related to the diffusion coefficient of lithium ions. The linear fit indicates that lithium-ion kinetics is significantly enhanced for Si TiGL 150 (*σ* = 58) compared to Si baseline (*σ* = 500) in agreement with the C-rate tests presented in Fig. 3c,d. A more detailed analysis was conducted by equivalent circuit fitting in order to extract the chemical diffusion coefficient (*D*_Li+_) of the electrodes. Figure 5c,d show impedance data measured at 50% SOC after the sample activation, and equivalent circuit for the fitting inset. The fitting results for both samples are presented in Table S3. The use of the Constant Phase Element (CPE) in parallel to the R_CT_ was motivated by the non-ideal response of the high-frequency semicircle together with a Warburg open (Wo) circuit element in series to the R_CT_-CPE circuit. Fits to the impedance data were satisfactory leading to low error % for the circuit elements used. Calculated *D*_Li+_ from fitted Wo-T^33^ indicate that lithium diffusivity is enhanced by four orders of magnitude; from 2.21 × 10^–12^  cm^2^ s^−1^ to 1.15 × 10^–8^ cm^2^ s^−1^ for Si baseline and Si TiGL 150, respectively. This is in good agreement with Fig. 4 showing an improved C-rate performance for the coated electrode. Indeed, diffusivity values herein presented are in good agreement to the reported ones for nano-silicon powder calculated by CV, GITT and EIS techniques and in the range of 10^–12^ cm^2^ s^−1^^34^. The improved performance of Si TiGL 150 delivering high discharge capacity 1200 mAh g^−1^ at 1 C and 500 mAh g^−1^ at 5 C is correlated to the enhanced electrode kinetics by titanicone coating. Electrochemical results for the optimized coated electrode are comparable to Si nanowires^35^ or nanotubes^36^ with Li diffusivities in the same order of magnitude e.g. 10^–8^ cm^2^ s^−1^.

## Discussion

TiGL coating provides an improvement in the performance of the nano-Si electrode as a negative electrode for LIBs. The performed analyses reveal that the optimum temperature to deposit TiGL over the silicon electrode is 150 °C, using 100 deposition cycles. These are optimized deposition parameters for the combined ALD/MLD process, an emergent technology to build next-generation batteries. Indeed, deposition techniques are spreading around battery industry as an efficient method working at sufficiently low temperatures to satisfy the integration requirements while maintaining reasonable throughput and cost. Here, the optimized electrode delivers 1800 mAh g^−1^ at 0.1 C and 500 mAh g^−1^ at 5 C, representing a substantial improvement compared to Si-based electrodes based on nano-metric powders. Moreover, the optimized TiGL coated Si electrode shows an outstanding capacity of 1200 mAh g^−1^ at 1 C for 350 cycles with capacity retention of 93%. The performance enhancement is directly correlated to the coating layer which acts as an interface modifier by improving efficiencies, discharge capacities and cycling stability. The coating layer boosts the electrical conductivity and lithium kinetics for diffusion by four orders of magnitude, leading to discharge capacities of 300 and 150 mAh g^−1^ at 10 C and 20 C rates, respectively.

## Methods

### Electrode preparation

Si nanoparticles (Alfa Aesar), Super P carbon black (TIMCAL) and polyvinylidene fluoride PVDF binder (Kynar HSV 900) were dried under vacuum overnight prior slurry formulation. PVDF and carbon black were first milled for 5 min and then dispersed uniformly in *N*-methyl-2-pyrrolidone (NMP) (99.5% Sigma Aldrich) by magnetic stirring, after that Si was added and the mixture stirred for 24 h to form a slurry. The slurry mass ratio was 60 wt% of Si NPs, 30 wt% carbon black, and 10 wt% PVDF. The final slurry was then casted onto a thin copper foil, using the doctor blade technique. After that, the electrodes were dried under vacuum at 80° and then pressed at 7 tons.

### ALD/MLD

TiCl_4_ (98% pure, Strem Chemicals Inc., U.S.A.), and glycerol (GL) (99.5% pure, Sigma-Aldrich, U.S.A.) were used as precursor and reactant, respectively, for electrode coating. TiCl_4_ was handled in a glovebox because of its air and moisture sensitivity. TiCl_4_ bottle was used at room temperature during the ALD/MLD process. The GL vessel was heated up to 60 °C. Nitrogen (N_2_) gas stream entrained the GL by flowing over the headspace in the GL vessel. Ultra-high purity grade N_2_ (99.999% pure, Airgas, CO, U.S.A.) was used to purge and carrier gas in the reactor. The ALD reactor used was ASM Microchemistry model F-120. The reactor base pressure was 1 Torr at an N_2_ gas flow rate of 100 sccm. The reactant pulse and N_2_ purge timing are designated as (t1, t2, t3, t4) where t1 is the TiCl_4_ exposure time, t2 is the purge after the TiCl_4_ exposure, t3 is the GL dose times, and t4 is the purge time after the GL exposures. The timing sequences were (3, 30, 3, 30 s), respectively, and the substrate was heated to different temperatures of 130 °C, 150 °C, 170 °C, 190 °C, and 210 °C. The process was repeated 100 times. Si electrodes with dimensions of 4 cm × 4 cm were used as a substrate. Table S1 in SI summarizes synthesis conditions for the samples and their nomenclature. In the paper, silicon coated samples are refereed as Si TiGL with their heating temperature e.g. Si TiGL 150.

### Cell assembly

Coin-type CR2032 cells were assembled in an Ar-filled glove box using a Li metal foil as a counter/reference electrode and the Si based electrodes as the working electrode. The electrolyte was 1 M LiPF_6_ dissolved in a 1:1 (volume ratio) mixture of ethylene carbonate (EC) and diethyl carbonate (DEC), the separator was a glass micro-filter (Whatman GF/F). The total mass loading of the active material in the electrode of diameter 14 mm was 1.3 mg.

### Characterization

X-ray diffraction (XRD) measurements were performed on a PANalytical X’Pert Pro MPD powder diffractometer with a Cu Kα X-ray source (λ = 0.154 nm). Scanning electron microscopy (SEM) images, along with energy-dispersive X-ray spectroscopy (EDX) measurements, were acquired using a JEOL model JSM-7500FA which was operated at 3 or 5 keV. The Transmission electron microscopy (TEM) used for the analysis was a JEOL JEM-2200FS and operated at 200 keV. A Fourier transform infrared (FTIR) ALPHA II Fourier-transform infrared (FTIR) spectrometer from Bruker was employed for infrared spectroscopy which was equipped with an Attenuated Total Reflection (ATR) module. The spectra were taken from 4000 to 400 cm^−1^ in absorbance mode. The electronic conductivity of electrodes was measured using a Jandel RM 3000 four points probe using currents between − 5 and 5 μA. For all these techniques, samples were transferred using a special sample holder that maintains vacuum conditions. In other words, the samples were not exposed to the atmosphere.

### Electrochemical measurement

Galvanostatic cycling was performed using a Neware BTS90000 Channel battery tester in the voltage range 0.1–2 V vs. Li/Li^+^ at different C-rates (0.10, 0.5, 0.2, 1, 2, 5, 10 and 20 C) assuming 1 C = 3579 mAh g^−1^. Prior rate-capability and capacity retention tests, electrodes were activated at 0.03 C for 2 cycle. Potentiostatic Electrochemical impedance spectroscopy (PEIS) measurements were conducted in the frequency range 20 kHz–0.1 Hz with an amplitude of 10 mV for the activated cells at 50% State of Charge (SOC). Impedance data was fitted to an equivalent circuit using the ZVIEW software.

## Supplementary Information

Supplementary Information.
