# Supplementary material for: High performance silicon electrode enabled by titanicone coating
Source: Sci Rep. 2022 Jan 7;12:137. doi: 10.1038/s41598-021-04105-x (PMC8741799; doi:10.1038/s41598-021-04105-x)
Supplement: Supplementary file 1 — Supplementary Information. [file 41598_2021_4105_MOESM1_ESM.pdf]

Supporting Information

High Performance Silicon Electrode Enabled by Titanicone Coating

Zahilia Cabán Huertas[1,2], Daniel Settipani [1], Cristina Flox[1], Joan Ramón Morante[2,3], Tanja Kallio[1*], and Jordi Jacas Biendicho[2*]

[1]Aalto University, Kemistintie 1, 02150 Espoo, Finland
[2]Catalonia Institute for Energy Research, Jardins de les Dones de Negre 1, 2ª p., 08930, Barcelona, Spain
[3]University of Barcelona, Faculty of Physics, Marti i Franques,1 Barcelona 08028 Spain
[*]Tanja Kalio tanja.kallio@aalto.fi Jordi Jacas Biendicho jjacas@irec.cat

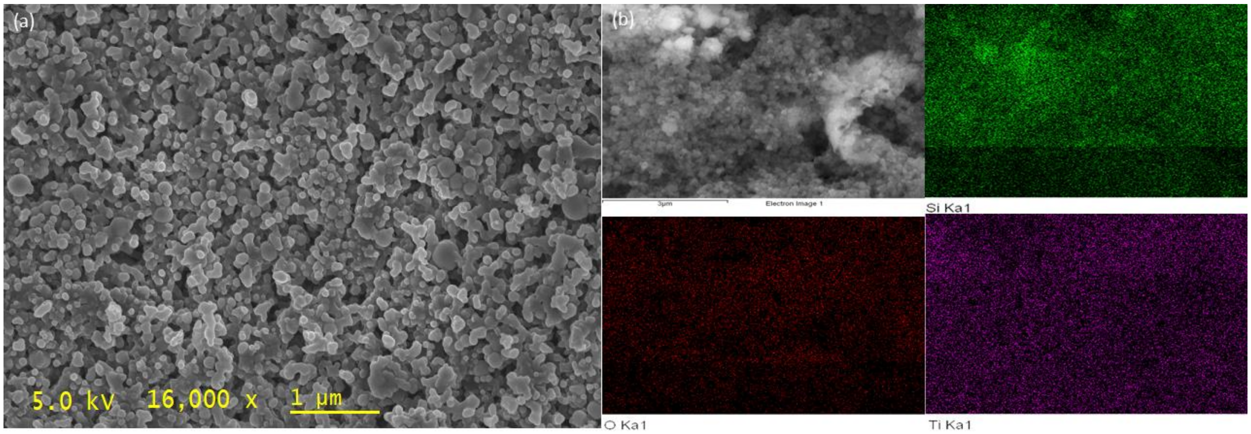

Figure S1. A SEM image and EDX mapping of Si TiGL 150 (Si green, O red, Ti purple).

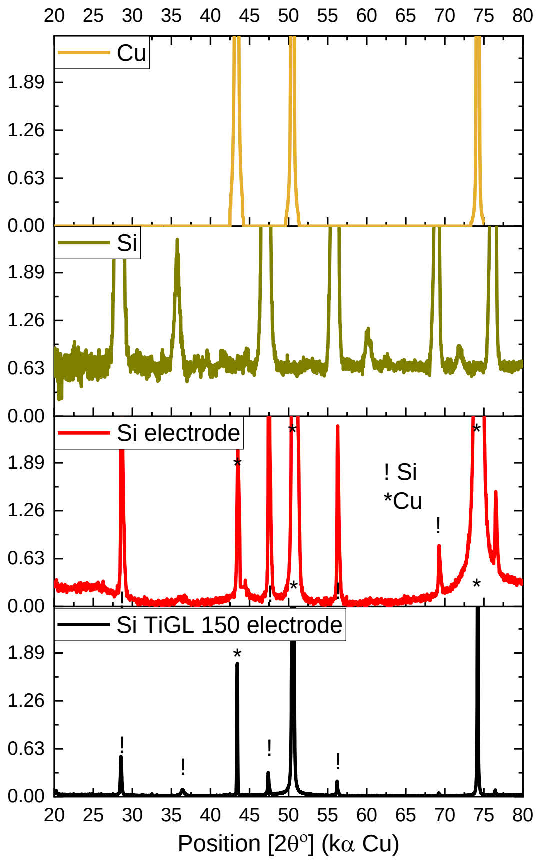

Figure S2. Power XRD of the Si and Si TiGL 150 electrode

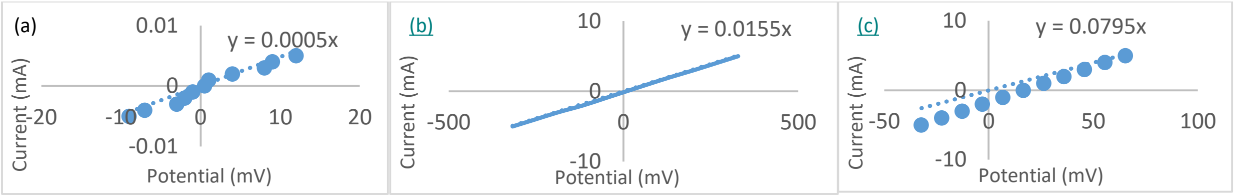

Figure S3 Current vs potential graphs of (a) TiGL over Si chip (b) Si baseline and (c) Si TiGL 150.

Name	Temperature ˚C	ALD/MLD cycles	Ti precursor deposition time (s)	GL deposition time (s)	Purge time (s)
Si baseline					
Si TiGL 130	130	100	3	3	30
Si TiGL 150	150	100	3	3	30
Si TiGL 170	170	100	3	3	30
Si TiGL 190	190	100	3	3	30
Si TiGL 210	210	100	3	3	30

Table S1. Nomenclature and titanicone synthesis conditions.

Name	Activation (1st cycle)	Activation (2st cycle)	Cycle at 0.10C (3rd cycle)	Activation (1st cycle)
Si baseline	27%	47%	57%	27%
Si TiGL 130	28%	54%	74%	28%
Si TiGL 150	47%	86%	98%	47%
Si TiGL 170	43%	81%	92%	43%
Si TiGL 190	46%	84%	95%	46%
Si TiGL 210	30%	50%	83%	30%

Table S2. CE% for un-coated and coated samples as-obtained for cycle 1 and 2 (electrode activation) and 0.10C cycling.

	Si baseline		Si TiGL 150	
	Value	Error %	Value	Error %
Rs / Ω	3.62	1.33	0.58	8.5
CPE1-T / µF	38	3.63	26	2.7
CPE1-P	0.81	0.55	0.84	0.36
Rct / Ω	101	1.38	30.95	0.51
W1-R / Ω	5240	10.58	7.75	3.53
W1-T / s	16.25	15.73	3.1×10^{-3}	11.82
W1-P	0.63	0.61	0.2	0.62
D_{Li} / cm^2s^{-1}	2.21×10^{-12}	-	1.15×10^{-8}	-

Table S3 Fitting results for Si baseline and Si TiGL 150 samples using equivalent circuit presented inset. For each element, error % are shown.

Lithium diffusivity was calculated using the most general form of the Warburg open circuit element whose impedance is defined as:

$$Z_w = R\frac{\coth(Tj\omega)^p}{(Tj\omega)^p}$$

where j= Wo-R, Wo-T and Wo-P are the fitting parameters and j=√(-1) and ω is angular frequency. The Wo-T fitted value can be correlated to the chemical diffusion coefficient or D_{chem} using formula below for which L is the diffusion length.

$$T = \frac{L^2}{D_{chem}}$$
